# Biomass Yield Efficiency of the Marine Anammox Bacterium, “*Candidatus* Scalindua sp.,” is Affected by Salinity

**DOI:** 10.1264/jsme2.ME14088

**Published:** 2015-02-13

**Authors:** Takanori Awata, Tomonori Kindaichi, Noriatsu Ozaki, Akiyoshi Ohashi

**Affiliations:** 1 EcoTopia Science Institute, Nagoya UniversityNagoya 464–8603Japan; 2 Department of Civil and Environmental Engineering, Graduate School of Engineering, Hiroshima UniversityHigashihiroshima 739–8527Japan

**Keywords:** anaerobic ammonium oxidation (anammox), *Candidatus* Scalindua, biomass yield efficiency, salinity, volatile fatty acids

## Abstract

The growth rate and biomass yield efficiency of anaerobic ammonium oxidation (anammox) bacteria are markedly lower than those of most other autotrophic bacteria. Among the anammox bacterial genera, the growth rate and biomass yield of the marine anammox bacterium “*Candidatus* Scalindua sp.” is still lower than those of other anammox bacteria enriched from freshwater environments. The activity and growth of marine anammox bacteria are generally considered to be affected by the presence of salinity and organic compounds. Therefore, in the present study, the effects of salinity and volatile fatty acids (VFAs) on the anammox activity, inorganic carbon uptake, and biomass yield efficiency of “*Ca.* Scalindua sp.” enriched from the marine sediments of Hiroshima Bay, Japan, were investigated in batch experiments. Differences in VFA concentrations (0–10 mM) were observed under varying salinities (0.5%–4%). Anammox activity was high at 0.5%–3.5% salinity, but was 30% lower at 4% salinity. In addition, carbon uptake was higher at 1.5%–3.5% salinity. The results of the present study clearly demonstrated that the biomass yield efficiency of the marine anammox bacterium “*Ca.* Scalindua sp.” was significantly affected by salinity. On the other hand, the presence of VFAs up to 10 mM did not affect anammox activity, carbon uptake, or biomass yield efficiency.

Anaerobic ammonium oxidation (anammox) is a microbiological process in which ammonium is oxidized to dinitrogen gas under anoxic conditions with nitrite as the electron acceptor (20, 31, 34). This process is mediated by anammox bacteria belonging to the phylum *Planctomycetes* (31). Previous studies demonstrated the ubiquitous distribution of anammox bacteria in artificial and natural ecosystems such as wastewater treatment plants, freshwater and marine sediments, and soils (2, 7, 8, 19, 24, 28, 29, 39). A minimum of five genera of candidate anammox bacteria have been tentatively proposed in *Planctomycetes*; *i.e.*, “*Candidatus* Brocadia,” “*Ca.* Kuenenia,” “*Ca.* Scalindua,” “*Ca.* Anammoxoglobus,” and “*Ca.* Jettenia” (9). “*Ca.* Scalindua” is primarily found in marine environments (16, 37), and 16S rRNA gene sequences have revealed that this genus contains taxonomically diverse members, even though only a few have been successfully grown in enrichment cultures to date (12, 14, 21, 36). Detailed, but partial information regarding some of the physiological characteristics of “*Ca.* Brocadia,” “*Ca.* Kuenenia,” and “*Ca.* Scalindua” are currently available (4, 6, 25, 26, 32), even though they have not yet been isolated in pure cultures.

Anammox bacteria are slow growing bacteria even under optimal growth conditions in laboratories with reported doubling times of 11 d for “*Ca.* Brocadia anammoxidans” (30), 8.3–11 d for “*Ca.* Kuenenia stuttgartiensis” (35), 7 d for “*Ca.* Brocadia sinica” (25), 14.4 d for “*Ca.* Scalindua sp.” (4), 3.6–5.4 d for anammox bacteria enriched from activated sludge and estimated by quantitative PCR (33), and 6–10 d for anammox bacteria enriched from a freshwater lake sediment and estimated by ^29^N_2_ measurements (38). These low growth rates have been supported by the following stoichiometry of the anammox process calculated by considering inorganic carbon fixation to cells (anabolism), as originally reported by Strous *et al.* (30):

$$1\;{{\text{NH}}_{4}}^{+}+1.32\;{{\text{NO}}_{2}}^{-}+0.066\;{{\text{HCO}}_{3}}^{-}+0.13\;{\text{H}}^{+}\to 1.02\;{\text{N}}_{2}+0.26\;{{\text{NO}}_{3}}^{-}+0.066\;{\text{CH}}_{2}{\text{O}}_{0.5}{\text{N}}_{0.15}+2.03\;{\text{H}}_{2}\text{O}$$

Using a membrane bioreactor, Lotti *et al.* (17) recently reported the following recalculated stoichiometry under no mass transfer limitations and no growth of non-anammox bacteria:

$$1\;{{\text{NH}}_{4}}^{+}+1.146\;{{\text{NO}}_{2}}^{-}+0.071\;{{\text{HCO}}_{3}}^{-}+0.057\;{\text{H}}^{+}\to 0.986\;{\text{N}}_{2}+0.161\;{{\text{NO}}_{3}}^{-}+0.071\;{\text{CH}}_{1.74}{\text{O}}_{0.31}{\text{N}}_{0.20}+2.002\;{\text{H}}_{2}\text{O}$$

Among the anammox genera, the growth rate of the marine genera “*Ca.* Scalindua” is markedly lower than that of the freshwater genera. This may be attributed to a lower inorganic carbon fixation (uptake) rate, leading to a lower biomass yield efficiency because the maximum specific ammonium consumption rate (65 μmol NH_4_^+^ [g protein]^−1^ min^−1^) of “*Ca.* Scalindua sp.” (4) is similar to that of freshwater anammox bacteria (25, 30, 31). In addition, the biomass yield efficiency of “*Ca.* Scalindua sp.” was determined to be 0.030 at 28°C and 3.5% salinity (4). Therefore, the differences observed in growth characteristics between freshwater and marine anammox bacteria raise questions regarding the influence of environmental factors.

We hypothesized that differences in the environmental concentrations of salt and certain organic compounds may affect the inorganic carbon uptake, biomass yield efficiency, and anammox activity of marine anammox bacteria. Therefore, the present study was conducted to determine the anammox activity and inorganic carbon uptake of the marine anammox bacterium, “*Ca.* Scalindua sp.” using batch experiments under different salinity and volatile fatty acid (VFA) concentrations. VFAs are also oxidized by both marine and freshwater anammox bacteria with nitrate as the electron acceptor (10, 11, 36). This metabolic variety may relate to a survival strategy of anammox bacteria in nutrient-limiting environments.

## Materials and Methods

### Biomass samples

Biomass samples were obtained from an upflow column reactor inoculated with an anammox biofilm, as previously described (14). The inoculant anammox biofilm contained two phylogenetically distinct “*Ca.* Scalindua sp.” operated at 20°C (14); therefore, the reactor was operated at 28°C to dominantly proliferate one species, as previously described (3, 4). The reactor volume was 920 mL and the hydraulic retention time was set to 4 h. The following slightly modified version of the synthetic marine nutrient medium reported by Kindaichi *et al.* (14) was fed into the reactor: 28 g L^−1^ of an artificial sea salt, SEALIFE (Marine Tech, Tokyo, Japan), 70 mg-N L^−1^ of (NH_4_ )_2_ SO_4_ , 70 mg-N L^−1^ of NaNO_2_ , 500 mg L^−1^ of KHCO_3_ , 27 mg L^−1^ of KH_2_ PO_4_ , 300 mg L^−1^ of MgSO_4_ ·7H_2_ O, 180 mg L^−1^ of CaCl_2_ ·2H_2_ O, and 1 mL of trace element solutions I and II, as described by van de Graaf *et al.* (34). The medium was flushed with N_2_ gas for 1 h to maintain an anaerobic condition (<0.5 mg L^−1^ of dissolved oxygen) before the addition of nutrients.

### Phylogenetic analysis

Total DNA was extracted from the upflow column reactor using the Fast DNA spin kit for soil (MP Biomedicals, Irvine, CA, USA) according to the manufacturer’s instructions. To construct the clone library, 16S rRNA gene fragments were amplified using the *Planctomycetales*-specific primer set of Pla46f (22) and 1390r (40). The PCR conditions were as follows: 4 min of initial denaturation at 94°C, followed by 30 cycles of 45 s at 94°C, 50 s at 58°C, and 3 min at 72°C. The final extension was performed for 10 min at 72°C. PCR products were confirmed using a 1% (w/v) agarose gel and were purified using the QIAquick PCR Purification Kit (Qiagen, Hilden, Germany). The purified PCR products were ligated into a pCR-XL-TOPO vector and transformed into One Shot *Escherichia coli* cells following the manufacturer’s instructions (TOPO XL PCR cloning kit; Invitrogen, Carlsbad, CA, USA). Cloned 16S rRNA genes were randomly selected and the clone library was constructed. The 16S rRNA genes were sequenced at the Dragon Genomics Center (Takara Bio, Otsu, Japan). Sequences with ≥97% homology were grouped into operational taxonomic units (OTUs) using the ARB neighbor-joining (NJ) method with similarity corrections performed using the ARB software (18). A phylogenetic tree was constructed using the ARB NJ (Felsenstein correction), maximum parsimony (Phylip DNAPARS), and maximum likelihood (RAxML) methods, which were implemented by the ARB software with a database SSU Ref NR 111 dataset (27). A bootstrap resampling analysis for 1,000 replicates was conducted to estimate the confidence of the tree topologies.

### Fluorescence *in situ* hybridization

Biomass samples were collected from the upflow column reactor and fixed in 4% paraformaldehyde. FISH was subsequently conducted according to the procedure described by Okabe *et al.* (23). The 16S rRNA-targeted oligonucleotide probes used in this study were EUBmix, composed of EUB338 (1), EUB338II, and EUB338III (5), which are specific for most bacteria; Amx368 (29), which is specific for all anammox bacteria; and Sca1129a, specific for uncultured bacterium husup-a2, and Sca1129b, specific for the uncultured bacterium husup-a7, which distinguish two phylogenetically different “*Ca.* Scalindua sp.” in the inoculum used in this study (14). The probes were labeled with Cy3 or Alexa Flour488 at the 5′ end. A LSM5 PASCAL model confocal laser-scanning microscope, equipped with an Ar ion laser (488 nm) and HeNe laser (543 nm; Carl Zeiss, Oberkochen, Germany), was used for microscopy. The average surface area fraction was determined from at least 24 representative laser scanning microscopy projection images using the LSM5 PASCAL software provided by Carl Zeiss (13).

### Batch experiments

Batch experiments were performed to investigate the influence of salinity (0.5%–4%) and VFAs (0–10 mM) on the anammox activity, inorganic carbon uptake, biomass yield efficiency, and VFA oxidation rates of “*Ca.* Scalindua sp.,” as summarized in Table 1. It should be noted that the batch experiment under 0% salinity conditions was not performed because of the lack of anammox activity observed in a previous study (4). The biomass from the upflow column reactor was washed twice in the synthetic marine nutrient medium without ammonium and nitrite and was then suspended in the synthetic marine nutrient medium containing 7 mM ammonium and nitrite at the volatile suspended solids (VSS) concentration of 1 mg VSS mL^−1^. A biomass suspension (4 mL) was dispensed into 7-mL serum vials and sealed with butyl rubber stoppers. The headspace was replaced by repeatedly vacuuming and purging with helium gas (>99.99995%). The vials were then statically incubated at 28°C for 1 d to determine the ammonium oxidation rate and inorganic carbon uptake and for 6 h to determine the VFA oxidation rate as described by Kartal *et al.* (10). All batch experiments were conducted in triplicate. Formate, acetate, and propionate were selected as organic compounds that can be oxidized by anammox bacteria as electron donors with nitrate as the electron acceptor, as previously reported (36). VFAs were added in the salt form (*i.e.*, sodium formate, sodium acetate, and sodium propionate) to prevent pH decreases by the addition of fatty acids. The pH was adjusted to 7.5 using 1 M NaOH or 1 M HCl after the addition of VFAs. [^14^C] bicarbonate was added to vials at a final concentration of 20 μCi (740 kBq vial^−1^). Anammox activity and inorganic carbon uptake were expressed as a specific ammonium consumption and [^14^C] bicarbonate uptake rate, respectively. The biomass yield efficiency was calculated by dividing the specific [^14^C]bicarbonate uptake rate by the specific ammonium consumption rate.

### Analytical methods

Ammonium concentrations were determined using Nessler’s method with a UV-visible spectrophotometer (DR-2800; Hach, Loveland, CO, USA). Nitrite and nitrate concentrations were determined using ion chromatography (HPLC 20A; Shimadzu, Kyoto, Japan) with a Shodex Asahipak NH2P-50 4D anion column (Showa Denko, Tokyo, Japan) and UV-VIS detector (SPD-20A; Shimadzu) following the filtration of samples through 0.2-μm pore size membranes (Advantec, Tokyo, Japan), as previously described (14). The concentration of VFAs was determined using HPLC (HPLC 20A; Shimadzu) with a Shim-packSCR-102H column (Shimadzu) and conductivity detector (CDD-10A vp; Shimadzu) following the filtration of samples through 0.2-μm pore size membranes (Advantec). [^14^C]bicarbonate uptake was confirmed by liquid scintillation counting. The biomass was collected, washed three times with phosphate buffered saline, and mixed with the scintillation cocktail (Clear-sol I; Nacalai Tesque, Kyoto, Japan). Radioactivity was subsequently determined using an LSC-5100 liquid scintillation counter (Hitachi-Aloka Medical, Tokyo, Japan).

### Statistical analysis

The average nitrogen stoichiometric ratios for consumed NO_2_^−^ and consumed NH_4_^+^ (ΔNO_2_^−^/ΔNH_4_^+^) and produced NO_3_^−^ and consumed NH_4_^+^ (ΔNO_3_^−^/ΔNH_4_^+^) in batch experiments for VFAs were compared using the unpaired Welch’s *t*-test in Microsoft Excel. A *P* value of less than 0.05 was considered significant.

### Nucleotide sequence accession number

The sequence data of the partial 16S rRNA gene obtained from the upflow column reactor was deposited in the GenBank/EMBL/DDBJ databases under accession number AB900163.

## Results and Discussion

### Dominant anammox species

**Fig. 1:**
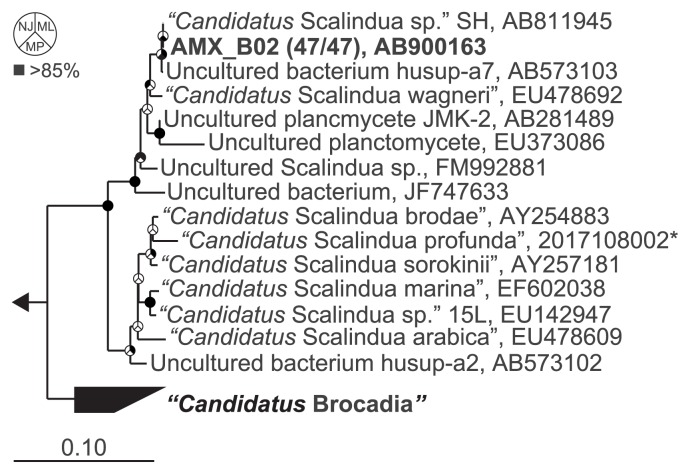
Maximum-likelihood (ML) tree of the “*Ca. Scalindua*” genus based on 16S rRNA gene sequences. The GenBank/EMBL/DDBJ accession numbers are indicated. The numbers in parentheses indicate the frequencies of identical clones analyzed. The scale bar represents the number of nucleotide changes per sequence position. Pie charts at the nodes represent the confidence of branch topology, and bootstrap values (1,000 replicates) greater than 85% are shaded black. The pie charts depict the neighbor-joining (NJ) method in the upper-left sector, ML method in the upper-right sector, and maximum parsimony (MP) method in the bottom sector. The asterisk represents the taxon ID in the Integrated Microbial Genomes with Microbiome Samples (IGM/M). The *Planctomyces brasiliensis* sequence (CP002546) served as the outgroup to root the tree.

To determine the dominant anammox species in the upflow column reactor, which had ammonium and nitrite removal efficiencies of 82.6% and 98.0%, respectively, a phylogenetic analysis on the basis of the 16S rRNA sequence was performed (Fig. 1). A total of 47 clones were randomly selected from the clone library and grouped into an OTU (AMX_B02) on the basis of ≥97% sequence identity. The sequence similarity between AMX_B02 and “*Ca.* Scalindua sp. SH” and the clone husup-a7 was 99.9%, indicating that a single species of “*Ca.* Scalindua sp.” was successfully enriched at 28°C, even though the inoculum contained two distinct species of anammox bacteria belonging to the “*Ca.* Scalindua” group (93.1% similarity to the clone husup-a2) (14). The dominance of a single species was further confirmed by a FISH analysis, which revealed that most anammox cells hybridized with the Amx368 (29) and Sca1129b (14) probes, whereas no cells hybridized with the Sca1129a probe (14). These results supported the findings of a previous study in which Sca1129b probe-hybridized cells dominated a reactor operated at 30°C (3). Anammox bacteria that were defined by Amx368 accounted for 73.5%±7.4% of all bacteria identified by the EUBmix probes. In the inoculum, Amx368 probe- and Sca1129a and Sca1129b probe-positive cells accounted for 60.8%, 17.3%, and 39.9% of all bacteria, respectively (14). The higher abundance of anammox bacteria in the present study may be related to the optimum growth temperature of “*Ca.* Scalindua sp.” because they have been shown to have higher anammox activities at 25°C–28°C than that at 30°C (4).

### Effects of salinity

**Fig. 2:**
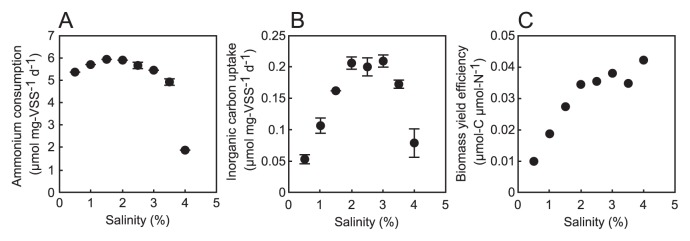
Effects of salinity on the specific ammonium consumption rate (A) and specific inorganic carbon uptake rate (B). The values in (A) and (B) are the mean standard deviations of the results of independent triplicate experiments. Biomass yield efficiency (C) is calculated by dividing the average specific inorganic carbon uptake rate by the average specific ammonium consumption rate.

The effects of 0.5%–4% salinity on the anammox activity of and carbon uptake by anammox bacteria were investigated in batch experiments. After the 1-d incubation, the simultaneous consumption of NH_4_^+^ and NO_2_^−^ was observed under all conditions. Higher anammox activities were observed at 0.5%–3.5% salinity, whereas anammox activity was lower at 4% salinity (approximately 30% of the highest activity at 1.5% salinity; Fig. 2A). Higher carbon uptake activity was also observed at 1.5%–3.5% salinity (Fig. 2B). The highest activity and carbon uptake values occurred at different salinity concentrations, *i.e.*, the highest activity was at 1.5%, but the highest carbon uptake was at 3% salinity, suggesting that the optimal conditions of “*Ca.* Scalindua sp.” differed with regard to anammox activity (catabolism) and carbon uptake (anabolism). Biomass yield efficiency was calculated to be 0.010–0.042 μmol-C μmol-N^−1^ under 0.5%–4.0% salinity conditions (Fig. 2C). The maximum growth rate at 3% salinity was calculated to be 0.00021 h^−1^; however, this value was markedly lower than the previous reported value (0.0020 h^−1^) (4) due to the batch incubation. It should be noted that the maximum value of 0.042 μmol-C μmol-N^−1^ at 4% salinity was not appropriate because anammox activity and carbon uptake were both low, and, thus, apparent biomass yield efficiency was high. Therefore, the average biomass yield efficiencies of 0.036 (in the range of 0.035–0.038) at 2–3% salinity, at which carbon uptake was high, were similar to previous findings: 0.07 for “*Ca.* Brocadia anammoxidans” (32), 0.063 for “*Ca.* Brocadia sinica” (25), and 0.030 for “*Ca.* Scalindua sp. SH” (4). The wide range of biomass yield efficiencies (in the range of 0.010–0.038) under ≤3.5% salinity conditions determined in this study indicated that the biomass yield efficiency of “*Ca.* Scalindua sp.” was strongly influenced by salinity. Under low salinity conditions (≤1%), biomass yield efficiencies (range, 0.010–0.019) were markedly lower than the previously reported value of 0.030 at 28°C and 3.5% salinity (4). In addition, a previous study showed that the biomass yield efficiency of “*Ca.* Scalindua sp. SH” was not affected by temperature differences (28°C and 37°C) (4). These results indicated that an appropriate salinity range (2%–3%) may be a key environmental factor for the growth of “*Ca.* Scalindua sp.” in estuary environments, which was originally collected as the inoculum for an anammox enrichment culture (14, 15). The biomass yield efficiencies of freshwater anammox bacteria also need to be estimated under various salinity conditions in future studies for comparisons between marine and freshwater species.

### Effects of volatile fatty acids

**Fig. 3:**
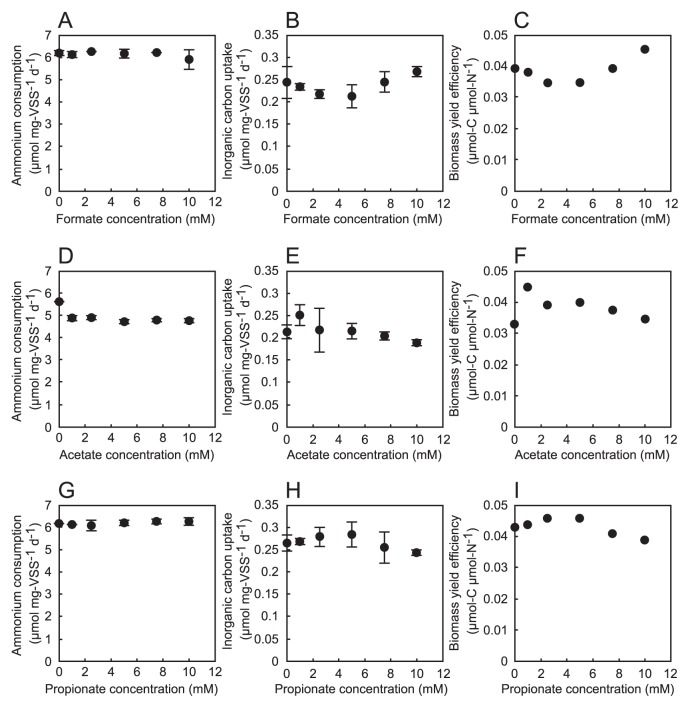
Effects of formate (A–C), acetate (D–F), and propionate (G–I) on the specific ammonium consumption rate (A, D, G) and specific inorganic carbon uptake rate (B, E, H). The values in (A, D, G) and (B, E, H) are the means ±SD of the results of independent triplicate experiments. Biomass yield efficiencies (C, F, I) were calculated by dividing the average specific inorganic carbon uptake rates by the average specific ammonium consumption rates.

To investigate the effects of VFAs on anammox activity and inorganic carbon uptake, formate, acetate, and propionate, all of which are known to be oxidized by anammox bacteria with nitrate as the electron acceptor (10, 11, 36), were selected and tested (Fig. 3). After the 1-d incubation, the simultaneous consumption of NH_4_^+^ and NO_2_^−^ was observed under all conditions. The average nitrogen stoichiometric ratios for ΔNO_2_^−^/ΔNH_4_^+^ and ΔNO_3_^−^/ΔNH_4_^+^ at 0 mM of formate, acetate, and propionate were 1.44 and 0.17, 1.20 and 0.03, and 1.34 and 0.08, respectively (Tables S1–S3). No significant differences were observed in these ratios (*P*>0.05) among the three VFAs. The average nitrogen stoichiometric ratios for ΔNO_2_^−^/ΔNH_4_^+^ and ΔNO_3_^−^/ΔNH_4_^+^ at 1–10 mM were then compared with the ratio at 0 mM for each VFA. The stoichiometric ratio was not significantly different (*P*>0.05) in any VFA experiment (Tables S1–S3). These results indicated that microbial processes other than anammox, such as VFA oxidation with nitrate and denitrification by co-existing bacteria in the biomass, were negligible. The average stoichiometric ratios in VFA experiments obtained in this study were similar to the previously reported ratios of marine anammox enrichment cultures, and were as follows: 1.22 and 0.22 (36), 1.28 and 0.24 (14), 1.21 and 0.15 (15), and 1.13 and 0.18 (26). In the case of formate and propionate, ammonium consumption did not change with increasing concentrations relative to ammonium consumption at 0 mM (Fig. 3A and G). On the other hand, ammonium consumption slightly decreased with the addition of acetate (Fig. 3D), although this decrease was not dose-dependent. No significant decrease in inorganic carbon uptake was observed, even though average values fluctuated. The average carbon uptake observed with the addition of formate, acetate, or propionate was in the range of 20% of the uptake at 0 mM (Fig. 3B, E, and H). Based on ammonium consumption and carbon uptake results, average biomass yield efficiencies under the addition of formate, acetate, or propionate were calculated to be 0.039, 0.038, or 0.043 μmol-C μmol-N^−1^, respectively (Fig. 3C, F, and I). These values were similar to the value of 0.030 under the 28°C and 2.8% salinity conditions reported by Awata *et al.* (4), but were 50% lower than the value reported for freshwater anammox bacteria (25, 32). One possible explanation for this lower yield efficiency is that “*Ca.* Scalindua spp.” require more energy for osmotic pressure adjustments or cell maintenance when salinity conditions are not optimal. However, future studies need to be conducted in order to confirm this phenomenon.

### VFA oxidation rates

Anammox bacteria are recognized as consumers of VFAs with nitrate as the electron acceptor (10, 11, 36). In the present study, the oxidation rates of VFAs of “*Ca.* Scalindua sp.” were similar to those of other anammox bacteria previously reported (Table 2). The VFA oxidation rates of “*Ca.* Scalindua sp.” in the present study and “*Ca.* Scalindua spp.” (36) were here than those of other freshwater anammox species, except for the acetate and propionate-consuming anammox bacteria “*Ca.* Brocadia fulgida” (11) and “*Ca.* Anammoxoglobus propionicus” (10). The higher VFA oxidation rates and tolerance to salinity of “*Ca.* Scalindua” species indicated that they had undergone niche adaptations to marine environments with lower ammonium and nitrite concentrations.

## Conclusion

The anammox activity, inorganic carbon uptake, and biomass yield efficiency of “*Ca.* Scalindua sp.” were determined by batch experiments under different salinities and VFA concentrations. We clearly demonstrated that the presence of VFAs did not affect these factors, whereas salinity significantly affected the carbon uptake of “*Ca.* Scalindua sp.” These results provide important new insights into the activity and growth characteristics of “*Ca.* Scalindua sp.” and indicate that salinity is one of the crucial factors regarding the nitrogen cycle in marine environments, especially at sites in which salinity fluctuates such as estuarine environments.

## Supplementary Information



## Figures and Tables

**Table 1:** Batch experimental conditions

Experiment*	NH_4_^+^ (mM)	NO_2_^−^ (mM)	NO_3_^−^ (mM)	Salinity (%)	VFA (mM)	[^14^C]bicarbonate (μCi vial^−1^)	Incubation time (h)
Salinity	7	7	0	0.5–4		0	24
	7	7	0	0.5–4		20	24
VFA	7	7	0	2.8	Formate, 0–10	0	24
	7	7	0	2.8	Formate, 0–10	20	24
	7	7	0	2.8	Acetate, 0–10	0	24
	7	7	0	2.8	Acetate, 0–10	20	24
	7	7	0	2.8	Propionate, 0–10	0	24
	7	7	0	2.8	Propionate, 0–10	20	24
VFA oxidation rate	7	7	4	2.8	Formate, 3	0	6
	7	7	4	2.8	Acetate, 3	0	6
	7	7	4	2.8	Propionate, 3	0	6

* All experiments were conducted in triplicate at 28°C with 1 mg-VSS mL^−1^.

**Table 2:** Volatile fatty acid (VFA) oxidation rates of different anaerobic ammonium oxidation (anammox) species

Species	Oxidation rate of VFA (μmol g-protein^−1^ min^−1^)			Reference

	Formate	Acetate	Propionate	
“*Candidatus* Brocadia anammoxidans”	6.5±0.6	0.57±0.05	0.12±0.01	(10)
“*Ca.* Brocadia fulgida”	7.6±0.6	0.95±0.04	0.31±0.007	(11)
“*Ca.* Kuenenia stuttgartiensis”	5.8±0.6	0.31±0.03	0.12±0.01	(10)
“*Ca.* Anammoxoglobus propionicus”	6.7±0.6	0.79±0.07	0.64±0.05	(10)
“*Ca.* Scalindua spp.”	7	0.7	0.3	(36)
“*Ca.* Scalindua sp.”	5.2±0.1	0.78±0.19	0.36±0.05	This study
