# Supplementary material for: Biomass Yield Efficiency of the Marine Anammox Bacterium, “Candidatus Scalindua sp.,” is Affected by Salinity
Source: Microbes Environ. 2015 Feb 13;30(1):86–91. doi: 10.1264/jsme2.ME14088 (PMC4356468; doi:10.1264/jsme2.ME14088)
Supplement: Supplementary file 1 [file 30_86_s1.pdf]

**Supplementary materials**

# Biomass yield efficiency of a marine anammox bacterium, "*Candidatus* Scalindua sp.," is affected by salinity

**Takanori Awata[1], Tomonori Kindaichi[2]*, Noriastu Ozaki[2], Akiyoshi Ohashi[2]**

*[1] EcoTopia Science Institute, Nagoya University, Nagoya 464-8603, Japan*

*[2] Department of Civil and Environmental Engineering, Graduate School of Engineering, Hiroshima University, Higashihiroshima 739-8527, Japan*

**Content:**

**Table S1**
**Table S2**
**Table S3**

Table S1 Nitrogen stoichiometric ratio in formate experiments

| Formate (mM) | Consumed $NH_4^+$ | Consumed $NO_2^-$ | Produced $NO_3^-$ |
|---|---|---|---|
| 0 | 1 | 1.47 | 0.18 |
| 0 | 1 | 1.43 | 0.16 |
| 0 | 1 | 1.43 | 0.16 |
| 1 | 1 | 1.50 | 0.17 |
| 1 | 1 | 1.43 | 0.16 |
| 1 | 1 | 1.45 | 0.16 |
| 2.5 | 1 | 1.44 | 0.15 |
| 2.5 | 1 | 1.42 | 0.15 |
| 2.5 | 1 | 1.41 | 0.14 |
| 5 | 1 | 1.44 | 0.14 |
| 5 | 1 | 1.42 | 0.13 |
| 5 | 1 | 1.50 | 0.15 |
| 7.5 | 1 | 1.45 | 0.13 |
| 7.5 | 1 | 1.43 | 0.13 |
| 7.5 | 1 | 1.44 | 0.13 |
| 10 | 1 | 1.47 | 0.13 |
| 10 | 1 | 1.66 | 0.14 |
| 10 | 1 | 1.43 | 0.12 |

Table S2 Nitrogen stoichiometric ratio in acetate experiments

| Acetate (mM) | Consumed $NH_4^+$ | Consumed $NO_2^-$ | Produced $NO_3^-$ |
|---|---|---|---|
| 0 | 1 | 1.09 | 0.01 |
| 0 | 1 | 1.11 | 0.01 |
| 0 | 1 | 1.39 | 0.07 |
| 1 | 1 | 1.25 | 0.01 |
| 1 | 1 | 1.29 | 0.03 |
| 1 | 1 | 1.25 | 0.01 |
| 2.5 | 1 | 1.24 | 0.02 |
| 2.5 | 1 | 1.27 | 0.01 |
| 2.5 | 1 | 1.28 | 0.01 |
| 5 | 1 | 1.31 | 0.01 |
| 5 | 1 | 1.31 | 0.00 |
| 5 | 1 | 1.29 | 0.01 |
| 7.5 | 1 | 1.29 | 0.01 |
| 7.5 | 1 | 1.31 | 0.01 |
| 7.5 | 1 | 1.28 | 0.00 |
| 10 | 1 | 1.31 | 0.00 |
| 10 | 1 | 1.32 | 0.00 |
| 10 | 1 | 1.27 | 0.00 |

Table S3 Nitrogen stoichiometric ratio in propionate experiments

| Propionate (mM) | Consumed $NH_4^+$ | Consumed $NO_2^-$ | Produced $NO_3^-$ |
|---|---|---|---|
| 0 | 1 | 1.34 | 0.09 |
| 0 | 1 | 1.33 | 0.08 |
| 0 | 1 | 1.35 | 0.08 |
| 1 | 1 | 1.36 | 0.07 |
| 1 | 1 | 1.34 | 0.05 |
| 1 | 1 | 1.35 | 0.06 |
| 2.5 | 1 | 1.32 | 0.02 |
| 2.5 | 1 | 1.34 | 0.02 |
| 2.5 | 1 | 1.43 | 0.02 |
| 5 | 1 | 1.36 | 0.00 |
| 5 | 1 | 1.32 | 0.00 |
| 5 | 1 | 1.32 | 0.00 |
| 7.5 | 1 | 1.34 | 0.00 |
| 7.5 | 1 | 1.33 | 0.00 |
| 7.5 | 1 | 1.31 | 0.00 |
| 10 | 1 | 1.29 | 0.00 |
| 10 | 1 | 1.31 | 0.01 |
| 10 | 1 | 1.36 | 0.00 |
